# Giant optical enhancement of strain gradient in ferroelectric BiFeO_3_ thin films and its physical origin

**DOI:** 10.1038/srep16650

**Published:** 2015-11-20

**Authors:** Yuelin Li, Carolina Adamo, Pice Chen, Paul G. Evans, Serge M. Nakhmanson, William Parker, Clare E. Rowland, Richard D. Schaller, Darrell G. Schlom, Donald A. Walko, Haidan Wen, Qingteng Zhang

**Affiliations:** 1 Advanced Photon Source, Argonne National Laboratory, Argonne, Illinois 60439, USA; 2 Department of Applied Physics, Stanford University, Stanford, CA 94305, USA; 3 Department of Materials Science and Engineering & Materials Science Program, University of Wisconsin–Madison, Madison, Wisconsin 53706, USA; 4 Department of Materials Science & Engineering, and Institute of Materials Science, University of Connecticut, Storrs, CT 06269-3136, USA; 5 Argonne Leadership Computing Facility, Argonne National Laboratory, Argonne, Illinois 60439, USA; 6 Department of Chemistry, Northwestern University, Evanston, IL 60208, USA; 7 Center for Nanoscale Materials, Argonne National Laboratory, Argonne, Illinois 60439, USA; 8 Department of Materials Science and Engineering, Cornell University, Ithaca, New York 14853, USA; 9 Kavli Institute at Cornell for Nanoscale Science, Ithaca, New York 14853, USA

## Abstract

Through mapping of the spatiotemporal strain profile in ferroelectric BiFeO_3_ epitaxial thin films, we report an optically initiated dynamic enhancement of the strain gradient of 10^5^–10^6^ m^−1^ that lasts up to a few ns depending on the film thickness. Correlating with transient optical absorption measurements, the enhancement of the strain gradient is attributed to a piezoelectric effect driven by a transient screening field mediated by excitons. These findings not only demonstrate a new possible way of controlling the flexoelectric effect, but also reveal the important role of exciton dynamics in photostriction and photovoltaic effects in ferroelectrics.

Flexoelectricity can be used to control the direction and magnitude of the spontaneous ferroelectric polarization using the electric field resulting from a strain gradient field, termed the flexoelectric field, pointing from high to low strain^1^. However, flexoelectric control of the polarization has been limited to static conditions because the strain gradients generated by strain relaxation^2^,^3^,^4^ in epitaxial thin films^4^,^5^,^6^,^7^,^8^ or by mechanical deformation^1^,^9^ cannot readily be dynamically modulated. The interaction of light with correlated materials has generated rich phenomena important for new material functionalities and for understanding the mechanisms governing these functionalities. Optical excitation of ferroelectric complex oxides, in particular, has generated a plethora of intriguing and potentially useful yet largely unexplained physical phenomena including photostriction^10^,^11^,^12^,^13^ and photovoltaic^14^,^15^ effects. Among these oxides, epitaxial multiferroic BiFeO_3_ (BFO)^16^ thin films have a strong structural and electronic response to excitation by photons with an energy larger than the direct band gap of 2.6–2.7 eV^17^. Absorption of these photons generates photo voltages larger than the bandgap^14^,^15^ and produces large lattice distortions^12^,^18^, neither of which are well understood due to the lack of information on the details of the structure and carrier dynamics. In addition, BFO epitaxial films also exhibit strain gradients larger than 10^5^ m^−1^ due to structural relaxation^3^ and a strong dependence of the polarization on flexoelectricity fields^7^,^8^.

Here we demonstrate that in BFO thin films the strain gradient can be significantly enhanced via ultrafast optical excitation by a magnitude comparable to the static strain gradient, i.e., 10^5^–10^6^ m^−1^, opening the possibility of direct coupling of flexoelectricity with optical stimuli. In addition, by correlating the structure and electronic dynamics for films with different thickness, we reveal the origin of the this strain gradient enhancement as a piezoelectric effect mediated by excitons, which also provides a new physical insight for photostriction and photovoltaic effects in ferroelectric thin films.

Both the optical and the X-ray measurements were performed under ambient conditions with the pump spot size bigger than the probe by a significant margin. Time-resolved X-ray diffraction (TRXRD) experiments were performed at beam line 7ID-C of the Advanced Photon Source. Optical excitation was provided by 50 fs laser pulses with a central wavelength of 400 nm, derived by frequency-doubling the output of a Ti:sapphire laser system that was synchronized to the X-ray pulses with an electronically adjustable time delay. Incident X-ray pulses with photon energies of 10 or 12 keV and pulse duration of 100 ps were used. The laser and the X-ray spot size are 700 μm and 50 μm in diameter, respectively. We used phase-pure epitaxial (0 0 1)-oriented BFO thin films of 4, 20, and 35 nm thickness grown directly on insulating SrTiO_3_ (STO) and (LaAlO_3_)_0.3_(Sr_2_AlTaO_3_)_0.7_ (LSAT) substrates by reactive molecular-beam epitaxy^17^. The high quality and phase purity of the samples were verified by X-ray reciprocal space mapping^19^ from which the domain sizes are estimated to be about 400, 40, and 25 nm for the 4, 20, and 35 nm thick films, respectively. The 4 nm film is tetragonal and the others are monoclinic^19^. Theory predicts that piezoelectricity disappears for BFO films thinner than 2 nm^20^. Experimentally, piezoelectric effects are reported for ultrathin BFO films down to 6 nm at 8 pm/V and drop to zero at 3 nm thickness^21^. Therefore, it is reasonable that a 4 nm film can still be weakly piezoelectric though it is not possible to directly measure the static piezoelectric effect for our samples due to the electrode-free configuration. The pump photon energy (400 nm, 3.1 eV) was below the band gaps of STO (3.2 eV) and LSAT (5.2 eV); thus, the photo response of the substrates was negligible in the measurement.

The real-space variation due to a strain gradient can be reconstructed from an analysis of the diffraction pattern^22^ in combination with knowledge of the orientation of the strain gradient expected from strain relaxation^23^. Our time-resolved diffraction measurement methods, with a schematic shown in Fig. 1(a), enable the reconstruction of a spatiotemporal map of the strain with a time resolution limited only by the X-ray pulse duration. The X-ray intensity along a crystal truncation rod cut through the (0 0 2) BFO Bragg peak at different delays between a pump laser pulse and a probe X-ray pulse is shown in Fig. 1(b) for a 35 nm film (More data are in the Supplementary Information (SI)). The strain derived from an analysis of the diffraction is reported in Fig. 1(c) relative to the average pseudo cubic lattice parameter of the film. The analysis method is described in the SI. The strain profiles for all time delays converge to ones that are dominated by a linear term (Fig. 1, SI Figs S1, S2, and S3) and can be approximated by (Fig. 1(b)):


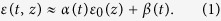


And the strain gradient is:


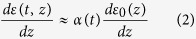


Here *ε*_0_ 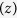 is the static strain profile before the arrival of the laser pulse, showing a relative compressive strain near the free surface and an expansion at the substrate-film interface (Fig. 1(c)), arising from relaxation of the epitaxial strain. In line with other studies^2^,^4^,^8^, the static strain gradients are 1.7×10^5^/m and 3.9×10^5^/m for the 35 and 20 nm films. The strain after the excitation is tilted by the factor α(*t*) with a shift of *β*(*t*). Direct comparison with the diffraction measurement identifies that the tilting factor α(*t*) is proportional to the overall spread of the lattice distortion that manifests itself into a broadening of the diffraction peak Δ*w*(*t*) (Fig. 1 (d)). The *β*(*t*) term, on the other hand, corresponds to the average transient strain Δ*ε* as derived from the angular shift of the Bragg peak (Fig. 1 (e)). Remarkably, upon the optical excitation at 3.3 mJ/cm^2^, the strain gradient increases to 3.0×10^5^/m and 1.0×10^6^/m for the 35 and 20 nm films, respectively (Fig. 1(d), Figs S2(b) and S3(b)).

To understand the mechanism driving the strain gradient change, the film thickness and pump fluence dependence was studied, where the broadening Δ*w* and the strain Δ*ε* of the (0 0 2) diffraction peak were measured as a function of the delay between the laser pump and the X-ray probe (Fig. 2). For a fixed film thickness, Δ*w* and Δ*ε* exhibit a nearly linear dependence on the fluence, thus exhibiting the same recovery dynamics. However, their peak value, i.e., the value immediately after the laser pulse, and the recovery time are found to be strongly dependent on the film thickness *Z*. While the recovery time is longer for thicker films, the peak Δ*w* and Δ*ε* are larger for thinner films (Fig. 2b,c) and inserts).

The transient absorption spectroscopy (TAS) experiment measures optical absorption of the sample as a function of the delay between the 40-fs, 400-nm pump laser pulse and the chirped 1 ps white-light probe laser pulse. The measured absorption spectrum has a wavelength ranging from 400 to 750 nm with time delays up to 7.2 ns. The relaxation time of the photo-induced absorption in TAS experiment, i.e., the photo-induced optical density (OD), also depends strongly on the film thickness (Fig. 3). The recovery times of the optical and structural relaxation are compared in Fig. 4. The epitaxial coherence of the samples excludes carrier trapping due to dislocations being the reason for the thickness dependence of the recovery time. It rather indicates that dynamics are determined by carrier diffusion and surface annihilation effect. A carrier diffusion coefficient *D* can be estimated^24^ by *τ*_*OD*_ = (*Z*/π)^2^/*D*^2^ 4, where Z is the sample thickness, giving an average diffusion coefficient *D* = 0.4 ± 0.05 nm^2^/ps.

The optically induced strain has been identified as arising from the piezoelectric effect^11^,^12^ due to the screening field formed by free carriers driven to the surface and interface by the internal polarization field. This model failed to explain the instantaneous onset of structure change^11^,^18^ due to the finite time required for the carriers to reach the surface and interface. To explain this instantaneous onset, a model based on lattice distortion by localized carriers arising from inhomogeneous photo-deposition has been proposed^18^.

However, the localized carrier model is inconsistent with the thickness dependence data. The photo-deposition as a function of the depth *z* follows an exponential function, i.e., exp(−*z/L*), where *L* = 32 nm is the BFO absorption length for 400 nm light. For thinner films, the photon deposition becomes more homogeneous and the expected strain spread and thus the diffraction peak broadening Δ*w* should reduce. This is contrary to our observation in Fig. 2 (b). Also, as the deposition profile induces a strain gradient with an opposite sign to the static strain gradient, the model also predicts a fluence dependent recovery with a narrowing of the diffraction peak when the photo-effect roughly cancels the intrinsic strain gradient. This is again not observed. In addition, the localization of the carriers also requires recovery dynamics independent of the film thickness, contrary to the data in Fig. 4. Our DFT simulation also rejects the notion of a strong carrier-lattice correlation (Fig. S4). Note that the normally important deformation potential effect^25^, as discussed previously^13^,^18^, due to the negative d*E*_g_/d*p*^26^,^27^, where *E*_g_ is the band gap and *p* is the pressure, causes lattice contraction and thus cannot be the main mechanism for the lattice expansion we observed here.

As BFO has a positive thermal expansion coefficient^28^ which is not a function of strain, any thermal contribution to the strain gradient can only be due to temperature gradient, i.e., higher at the free surface and lower at the interface, which has an opposite sign to the observed strain gradient. Therefore, we conclude that thermal effects do not contribute to the enhancement of the strain gradient.

To construct a physical model consistent with the experiment observation, we note that the distortion of the lattice in ferroelectric material arising from an electric field is


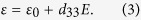


Though generally regarded as a constant, the macroscopic piezoelectric coefficient *d*_33_ is dependent on the applied field^29^ and strain states^30^ in epitaxial films. Thus Eq. (1) can be rewritten as


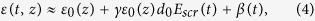


with


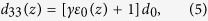



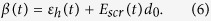


Here, γ represents the first-order dependence of the piezoelectric coefficient on the strain and *d*_0_ is the average piezoelectric coefficient of the film; *E*_scr_ and *ε*_h_ are the time-dependent screening field and the thermal contribution to the strain. Using the experimental data, we have γ = 220 ± 75 for the 35 nm film and γ = 160 ± 50 for the 20 nm film (see SI Table S1). These values are higher than γ = 40 ± 60 inferred from the data between STO and LSAT substrates from Daumont *et al.*^30^. Given the differences in samples and measurement method, this is not unexpected.

Note that in Eq. (4), the screening field *E*_scr_ is spatially homogeneous, requiring negligible presence of free carriers within the film. Thus the laser-excited carriers must be dominantly charge-neutral entities, i.e., excitons. These excitons dissociate and generate free carriers at the surface and interface by local band bending^31^,^32^. The free carriers then either stay or migrate to the other side of the film depending on their charge sign, the local polarization, and the screening field. Direct separation of the charge carriers by the internal polarization field, in contrast, can cause a dramatic field distortion in the film yielding strain profiles different form those measured and are thus incompatible with our data (see Figs S5 and S6).

It has been pointed out that formation of self-trapped charge *p-d* transfer excitons, i.e., with a hole in the O-*p* and an electron in the B site metal *d* orbitals, occurs in BFO^33^ and other perovskite ferroelectrics^34^, an observation deduced from extensive analysis of the structureless spectral band below the bandgap such as these in Fig 3. Similar spectra are also reported by other authors for BFO^33^,^35^,^36^. While further theoretical worked is needed to understand the details regarding the electronic structure and the hopping dynamics of these excitons, our X-ray measurement showing the neutrality of the photo-excited carriers is a solid confirmation of the excitonic interpretation of the spectral feature. Note that the correlated lattice distortion of the excitons as proposed in literature^34^ does not lead to observable lattice effects in our experiment.

This exciton-based model is consistent with all experimental observations. The photoexcitation steepens the existing strain gradient via dependence of the piezoelectric coefficient on the strain. As the screening field is inversely proportional to the film thickness, the effect is more pronounced for thinner films, thus increasing the broadening Δ*w*. Dissociation of excitons located at or adjacent to the surface and interface lead to the instantaneous onset of the structural change while the carriers inside the film must diffuse to the surface and interface to dissociate, leading to thickness-dependent dynamics (see the schematic in Fig. 5). Note that as the direction of the native polarization, the depolarization field and the screening field are self-consistently aligned, screening of the depolarization field always leads to expansion of the lattice and thus positive strain.

The giant enhancement of the strain gradient in ferroelectrics has significant practical implications. It is useful to estimate the magnitude of the static and the dynamic flexoelectric effect in our samples. For the 35 nm BFO thin film, in which the static strain gradient is on the order of 2×10^5^ m^−1^, the steady state flexoelectric field is estimated to be 9 MV m^−1^ which in large part determines the polarization of as-grown films^8^. Doubling or tripling the strain gradient will increase the flexoelectric field to approximately 20 MV m^−1^, close to the coercive field for most epitaxial ferroelectric thin films^37^. Note that the difference between the tetragonal and monoclinic symmetry is that the ferroelectric polarization is along (0 0 1) in the former and (1 1 1) in the latter. The flexoelectric effect, however, is only determined by the orientation of the strain gradient; thus the crystal symmetry plays a minor role in determining the flexoelectric effect. As strain can be engineered independently from the polarization, applications such as direct optical writing of non-volatile ferroelectric memory^38^ thus become possible using the dynamically induced flexoelectric effect. It is also possible now to manipulate the strain gradient without the need of physical deformation^9^, opening new horizons for studying dynamic flexoelectric effects in nanoscale devices.

Our results clearly reveal the physical origin of the photostriction effect in ferroelectric BFO thin films, especially the importance of the dynamics of the excitons. The experiment is also effectively an electrode-free photovoltaic experiment that maps the field distribution and the charge carrier dynamics in the thin film; thus the derived exciton-based model provides a new insight that significantly deviates from the existing bulk photovoltaic effect theory^15^,^39^,^40^,^41^ for ferroelectric photovoltaic effects. The method of correlating the carrier dynamics and the strain profile may find wider applications in understanding complex oxide thin film systems where the functionality is strongly dependent on the correlation between structural and other degrees of freedom. Our electrode-free samples are incompatible with relevant c-axis transport measurements which require a conductive layer such as La_0.67_Sr_0.33_MnO_3_ between the substrate and the film. In the future it would be very useful to be able to relate the carrier dynamics inferred from our data to a direct transport measurement.

## Additional Information

**How to cite this article**: Li, Y. *et al.* Giant optical enhancement of strain gradient in ferroelectric BiFeO_3_ thin films and its physical origin. *Sci. Rep.* **5**, 16650; doi: 10.1038/srep16650 (2015).

## Supplementary Material

Supplementary Information

## Figures and Tables

**Figure 1:**
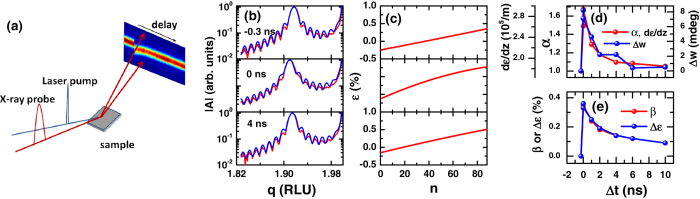
(**a**) Schematic of the time-resolved X-ray diffraction experiment. (**b**) Diffraction amplitude |A| along the truncation rod cut through the (0 0 2) diffraction peak for a 35 nm BFO thin film at a nominal fluence of 3.3 mJ/cm^2^ for delays of −0.3, 0, and 4 ns (red) experimental data, (blue) fit. Reciprocal Lattice Units (RLU) are with respect to the STO substrate. (**c**) Corresponding strain as a function of the monolayer index. More data are in Figs S1–S3. (**d**) Parameters *α* and the corresponding strain gradient *dε*/*dz* in Eqs (1) as a function of delay between the X-ray and the laser Δ*t* (red) in comparison with measured width change Δ*w* (blue). (**e**) Parameter *β* in Eq. (1) (red) and the measured average strain change Δ*ε* (blue).

**Figure 2:**
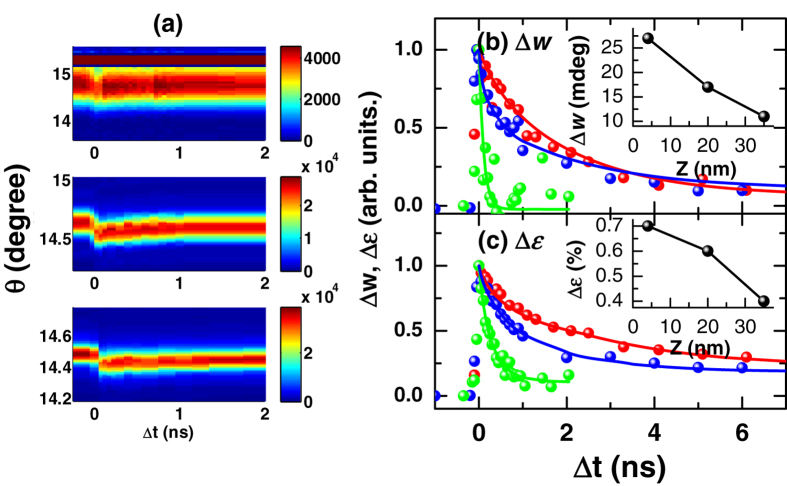
(**a**) X-ray diffraction near the (0 0 2) Bragg peak in BFO films as a function of Δ*t* with film thicknesses *Z* = 4 nm (top), 20 nm (center) and 35 nm (bottom), taken at a nominal laser fluence of 2.5 mJ cm^−2^. (**b**) Normalized broadening of the diffraction peak Δ*w* and (**c**) average strain Δ*ε* for Z = 4 (green), 20 (blue), and 35 nm (red), with their corresponding peak value at Δ*t* = 0 as a function of *Z* in the inserts. Solid lines are stretched exponential fits to the strain (see Fig. 4 caption). The intense feature at 15.2° in the top panel in (**a**) is the (0 0 2) Bragg peak from the STO substrate.

**Figure 3:**
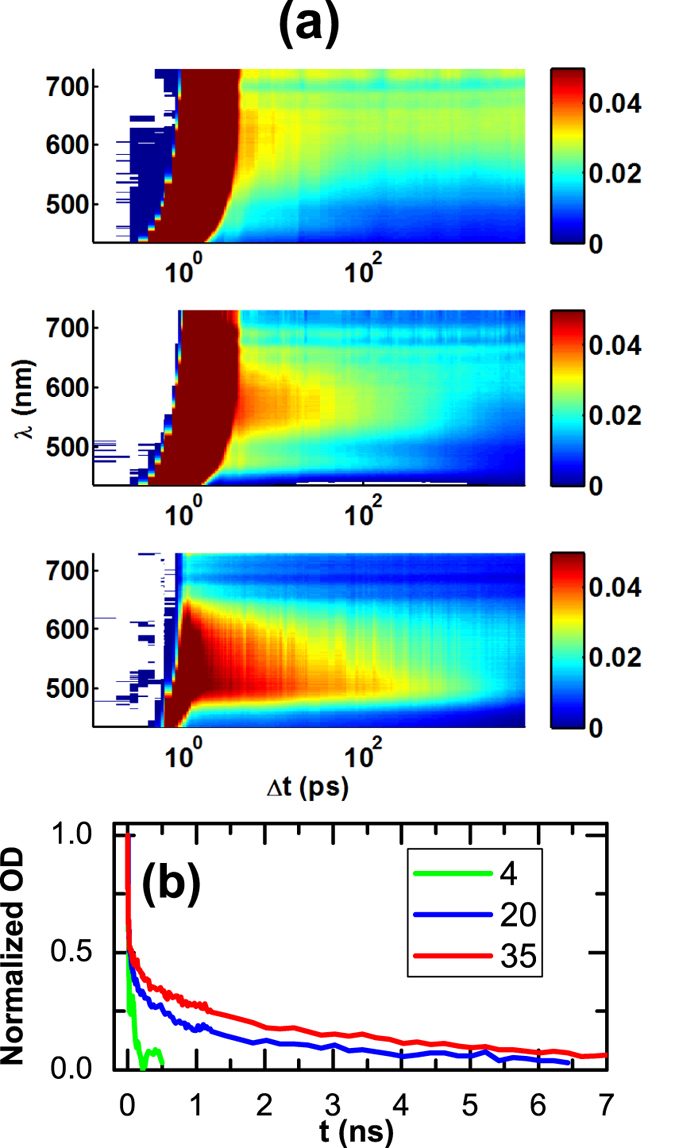
(**a**) Net change of the absorption spectra as a function of delay (Δ*t*) between the pump and the probe taken at nominal fluence of 5.5, 5.5, and 4.7 mJ/cm^2^ respectively for (from top to bottom) Z = 4, 20 and 35 nm films. (**b**) Normalized change in OD as a function of delay for the three film thickness integrated in the wavelength range 490–600 nm.

**Figure 4:**
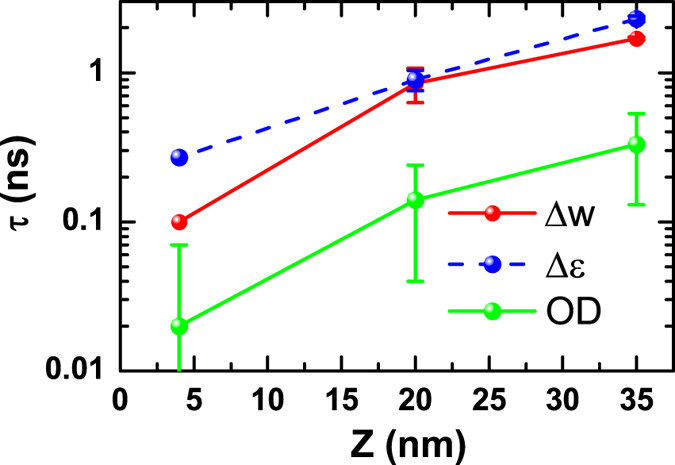
Recovery time for the relaxation of optical density (OD) and average strain (*∆ε*) and diffraction peak broadening (*∆w*). The 1/e recovery time τ is extracted by fitting to a stretched exponential function, *f*(*t*) = *a* + *b* exp(−(*t*/τ)^η^), with *a*, *b* and η being fitting parameters.

**Figure 5:**
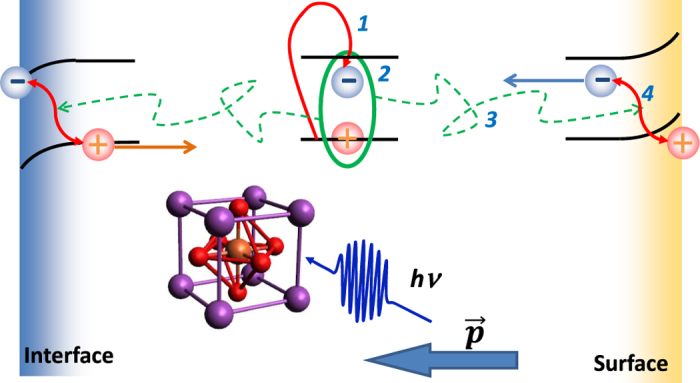
Schematic of the carrier dynamics leading to the steepening of the strain gradient. (1) Electrons are excited and (2) cool to form excitons. (3) Excitons then diffuse to the interface and surface where they (4) dissociate due to local band bending. The free carriers screen the depolarization field leading to a structural piezoelectric response. The strain dependence of the piezoelectric coefficient leads to the steepening of the static strain gradient. A BFO unit cell is shown in the inset with Bi, Fe, and O atoms represented by purple, brown, and red balls. Note that the initial film polarization direction has minimal effect on the lattice change.

## References

[b1] Zubko P., Catalan G. & Tagantsev A. K. Flexoelectric Effect in Solids. Annu. Rev. Mater. Res. 43, 387–421 (2013).

[b2] Catalan G., Noheda B., McAneney J., Sinnamon L. & Gregg J. Strain gradients in epitaxial ferroelectrics. Phys. Rev. B 72, 020102 (2005).

[b3] Zeches R. J. *et al.* A Strain-Driven Morphotropic Phase Boundary in BiFeO_3_. Science 326, 977–980 (2009). 19965507 10.1126/science.1177046

[b4] Lee D. *et al.* Giant Flexoelectric Effect in Ferroelectric Epitaxial Thin Films. Phys. Rev. Lett. 107, 057602 (2011). 21867099 10.1103/PhysRevLett.107.057602

[b5] Catalan G. *et al.* Flexoelectric rotation of polarization in ferroelectric thin films. Nat. Mater. 10, 963–967 (2011). 22001961 10.1038/nmat3141

[b6] Lee D., Yang S. M., Yoon J.-G. & Noh T. W. Flexoelectric Rectification of Charge Transport in Strain-Graded Dielectrics. Nano Lett. 12, 6436–6440 (2012). 23190011 10.1021/nl3038129

[b7] Borisevich A. Y. *et al.* Atomic-scale evolution of modulated phases at the ferroelectric–antiferroelectric morphotropic phase boundary controlled by flexoelectric interaction. Nat. Commun. 3, 775 (2012). 22491323 10.1038/ncomms1778

[b8] Jeon B. C. *et al.* Flexoelectric effect in the reversal of self-polarization and associated changes in the electronic functional properties of BiFeO_3_ thin films. Adv. Mater. 25, 5643–5649 (2013). 23897638 10.1002/adma.201301601

[b9] Lu H. *et al.* Mechanical Writing of Ferroelectric Polarization. Science 336, 59–61 (2012). 22491848 10.1126/science.1218693

[b10] Kundys B., Viret M., Colson D. & Kundys D. O. Light-induced size changes in BiFeO_3_ crystals. Nat. Mater. 9, 803–805 (2010). 20657588 10.1038/nmat2807

[b11] Daranciang D. *et al.* Ultrafast Photovoltaic Response in Ferroelectric Nanolayers. Phys. Rev. Lett. 108, 087601 (2012). 22463572 10.1103/PhysRevLett.108.087601

[b12] Wen H. *et al.* Electronic Origin of Ultrafast Photoinduced Strain in BiFeO_3_. Phys. Rev. Lett. 110, 037601 (2013). 23373952 10.1103/PhysRevLett.110.037601

[b13] Lejman M. *et al.* Giant ultrafast photo-induced shear strain in ferroelectric BiFeO_3_. Nat. Commun. 5, 4301 (2014). 24980954 10.1038/ncomms5301

[b14] Yang S. Y. *et al.* Above-bandgap voltages from ferroelectric photovoltaic devices. Nat. Nanotechnol. 5, 143–147 (2010). 20062051 10.1038/nnano.2009.451

[b15] Bhatnagar A., Roy Chaudhuri A., Heon Kim Y., Hesse D. & Alexe M. Role of domain walls in the abnormal photovoltaic effect in BiFeO_3_. Nat. Commun. 4, 1 (2013).

[b16] Wang J. Epitaxial BiFeO_3_ Multiferroic Thin Film Heterostructures. Science 299, 1719–1722 (2003). 12637741 10.1126/science.1080615

[b17] Ihlefeld J. F. *et al.* Optical band gap of BiFeO_3_ grown by molecular-beam epitaxy. Appl. Phys. Lett. 92, 142908 (2008).

[b18] Schick D. *et al.* Localized Excited Charge Carriers Generate Ultrafast Inhomogeneous Strain in the Multiferroic BiFeO_3_. Phys. Rev. Lett. 112, 097602 (2014). 24655276 10.1103/PhysRevLett.112.097602

[b19] Yang Y., Schlepütz C. M., Adamo C., Schlom D. G. & Clarke R. Untilting BiFeO_3_: The influence of substrate boundary conditions in ultra-thin BiFeO_3_ on SrTiO_3_. APL Mater. 1, 052102 (2013).

[b20] Jiang Q. & Qiu J. H. The thickness dependence of ferroelectric and magnetic properties in epitaxial BiFeO_3_ thin films. J. Appl. Phys. 99, 103901 (2006).

[b21] Zhao J. L., Lu H. X., Sun J. R. & Shen B. G. Thickness dependence of piezoelectric property of ultrathin BiFeO_3_ films. Phys. B Condens. Matter 407, 2258–2261 (2012).

[b22] Newton M. C., Leake S. J., Harder R. & Robinson I. K. Three-dimensional imaging of strain in a single ZnO nanorod. Nat. Mater. 9, 120–124 (2009). 20023632 10.1038/nmat2607

[b23] Kwak B. *et al.* Strain relaxation by domain formation in epitaxial ferroelectric thin films. Phys. Rev. Lett. 68, 3733–3736 (1992). 10045783 10.1103/PhysRevLett.68.3733

[b24] Sproul A. B. Dimensionless solution of the equation describing the effect of surface recombination on carrier decay in semiconductors. J. Appl. Phys. 76, 2851 (1994).

[b25] Thomsen C., Grahn H., Maris H. & Tauc J. Surface generation and detection of phonons by picosecond light pulses. Phys. Rev. B 34, 4129–4138 (1986). 10.1103/physrevb.34.4129 9940178

[b26] Gavriliuk A. *et al.* Another mechanism for the insulator-metal transition observed in Mott insulators. Phys. Rev. B 77, 155112 (2008).

[b27] Gómez-Salces S. *et al.* Effect of pressure on the band gap and the local FeO_6_ environment in BiFeO_3_. Phys. Rev. B 85, 144109 (2012).

[b28] Catalan G. & Scott J. F. Physics and Applications of Bismuth Ferrite. Adv. Mater. 21, 2463–2485 (2009).

[b29] Nagarajan V. *et al.* Realizing intrinsic piezoresponse in epitaxial submicron lead zirconate titanate capacitors on Si. Appl. Phys. Lett. 81, 4215 (2002).

[b30] Daumont C. *et al.* Strain dependence of polarization and piezoelectric response in epitaxial BiFeO_3_ thin films. J. Phys. Condens. Matter 24, 162202 (2012). 22467186 10.1088/0953-8984/24/16/162202

[b31] Pintilie L. & Alexe M. Metal-ferroelectric-metal heterostructures with Schottky contacts. I. Influence of the ferroelectric properties. J. Appl. Phys. 98, 124103 (2005).

[b32] Apostol N. G. *et al.* Band bending at free Pb(Zr,Ti)O_3_ surfaces analyzed by X-ray photoelectron spectroscopy. Mater. Sci. Eng. B 178, 1317–1322 (2013).

[b33] Pisarev R., Moskvin A., Kalashnikova A. & Rasing T. Charge transfer transitions in multiferroic BiFeO_3_ and related ferrite insulators. Phys. Rev. B 79, 235128 (2009).

[b34] Eglitis R., Kotomin E., Trepakov V., Kapphan S. & Borstel G. Quantum chemical modelling of electron polarons and’green’luminescence in PbTiO_3_ perovskite crystals. J. Phys. Condens. Matter 14, L647 (2002).

[b35] Sheu Y. M. *et al.* Ultrafast carrier dynamics and radiative recombination in multiferroic BiFeO_3_. Appl. Phys. Lett. 100, 242904 (2012).

[b36] Yamada Y., Nakamura T., Yasui S., Funakubo H. & Kanemitsu Y. Measurement of transient photoabsorption and photocurrent of BiFeO_3_ thin films: Evidence for long-lived trapped photocarriers. Phys. Rev. B 89, 035133 (2014).

[b37] Dawber M. & Scott J. F. Physics of thin-film ferroelectric oxides. Rev. Mod. Phys. 77, 1083–1130 (2005).

[b38] Guo R. *et al.* Non-volatile memory based on the ferroelectric photovoltaic effect. Nat. Commun. 4, 1990 (2013). 23756366 10.1038/ncomms2990 PMC3709492

[b39] Glass A. M. High-voltage bulk photovoltaic effect and the photorefractive process in LiNbO_3_. Appl. Phys. Lett. 25, 233 (1974).

[b40] Fridkin V. M. Bulk photovoltaic effect in noncentrosymmetric crystals. Crystallogr. Rep. 46, 654–658 (2001).

[b41] Ji W., Yao K. & Liang Y. C. Bulk Photovoltaic Effect at Visible Wavelength in Epitaxial Ferroelectric BiFeO_3_ Thin Films. Adv. Mater. 22, 1763–1766 (2010). 20496412 10.1002/adma.200902985

