# Supplementary material for: Giant optical enhancement of strain gradient in ferroelectric BiFeO3 thin films and its physical origin
Source: Sci Rep. 2015 Nov 20;5:16650. doi: 10.1038/srep16650 (PMC4653733; doi:10.1038/srep16650)
Supplement: Supplementary Information [file srep16650-s1.pdf]

`

Giant optical enhancement of strain gradient in ferroelectric $BiFeO_3$ thin films and its physical origin

Yuelin Li[1], Carolina Adamo[2], Pice Chen[3], Paul G. Evans[3],

Serge M. Nakhmanson[4], William Parker[5], Clare E. Rowland[6], Richard D. Schaller[7],

Darrell G. Schlom[8,9], Donald A. Walko[1], Haidan Wen[1], and Qingteng Zhang[3]

[1] Advanced Photon Source, Argonne National Laboratory, Argonne, Illinois 60439, USA

[2] Department of Applied Physics, Stanford University, Stanford, CA 94305, USA

[3] Department of Materials Science and Engineering & Materials Science Program, University of Wisconsin–Madison, Madison, Wisconsin 53706, USA

[4]Department of Materials Science & Engineering, and Institute of Materials Science University of Connecticut, Storrs, CT 06269-3136, USA

[5] Argonne Leadership Computing Facility, Argonne National Laboratory, Argonne, Illinois 60439, USA

[6] Department of Chemistry, Northwestern University, Evanston, IL 60208, USA

[7] Center for Nanoscale Materials, Argonne National Laboratory, Argonne, Illinois 60439, USA

[8] Department of Materials Science and Engineering, Cornell University, Ithaca, New York 14853, USA

[9] Kavli Institute at Cornell for Nanoscale Science, Ithaca, New York 14853, USA

[1] Correspondence should be sent to Yuelin Li: ylli@aps.anl.gov

`

Strain profile retrieval methods

The diffraction amplitude from a thin film with the contribution from the substrate is calculated using a kinetic diffraction model,

$$|A_{fit}(q)| = \left|FFT[\exp(i\varphi_n)]F_{BFO}(q) + \frac{aF_{STO}(q)}{[1-\exp(iq)]}\right|, \qquad (s1)$$

Here φ is the layer-by-layer BFO phase shift of the unit cells, F_{BFO} and F_{STO} are the structure factors, *n=1,…, N* and is the layer index and *N* is the number of epitaxial layers, and *a* is the fitting parameter for the relative intensity of the substrate and the film, respectively.

The fitting of the strain profile takes our knowledge of the film thickness and is accomplished by a spline algorithm[1] using 4 evenly distributed fitting points for adjusting the phase while using a spline interpolation to obtain the phase at each unit cell layer. Fitting with more points does not change the fitting qualitatively and only reduce the fitting error modestly by less than 10%. The fitting algorithm tries to minimize the following error while changing the phase:

$$\sum_q ||A_{fit}(q)| - |A_{mea}(q)|| \ , \qquad (s2)$$

where $|A_{mea}(q)|=|I(q)|^{1/2}$ is the measured diffraction amplitude along the truncation rod.

The measurement was repeated at different delays for samples with different laser fluences and film thicknesses. A complete set of data is depicted in Figs. S1-S3. The fitting matches the position and the relative amplitude of the fringes well except for the 20 nm film at time zero, which needs further study to determine the cause.

`

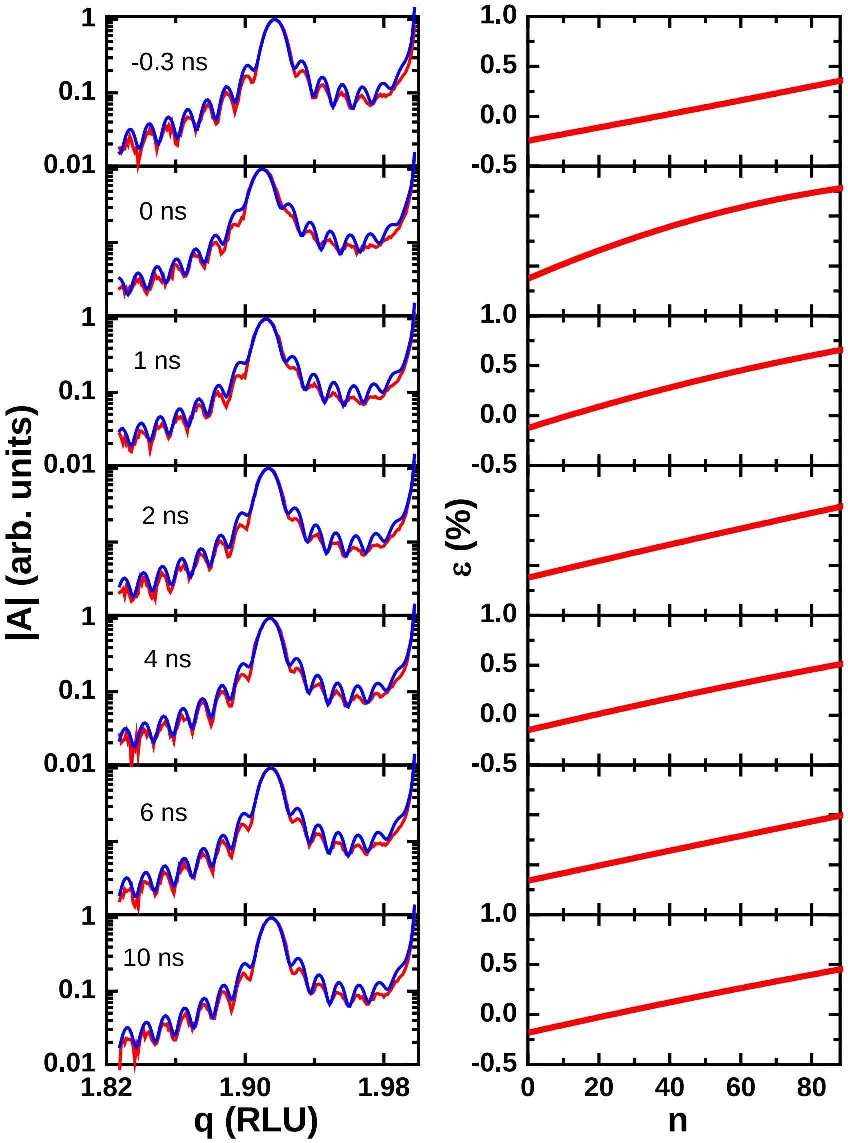

Figure S1 A complete data set of the mapping of the strain as a function of delay for a 35 nm film (88 monolayers) at 3.3 mJ/cm^2. Left column: measured (red lines) and fitted (blue lines) diffraction amplitude |A| at different delays between the laser and the X-ray. Left column: corresponding fitted strain at different delays as a function of the film layer index *n*.

`

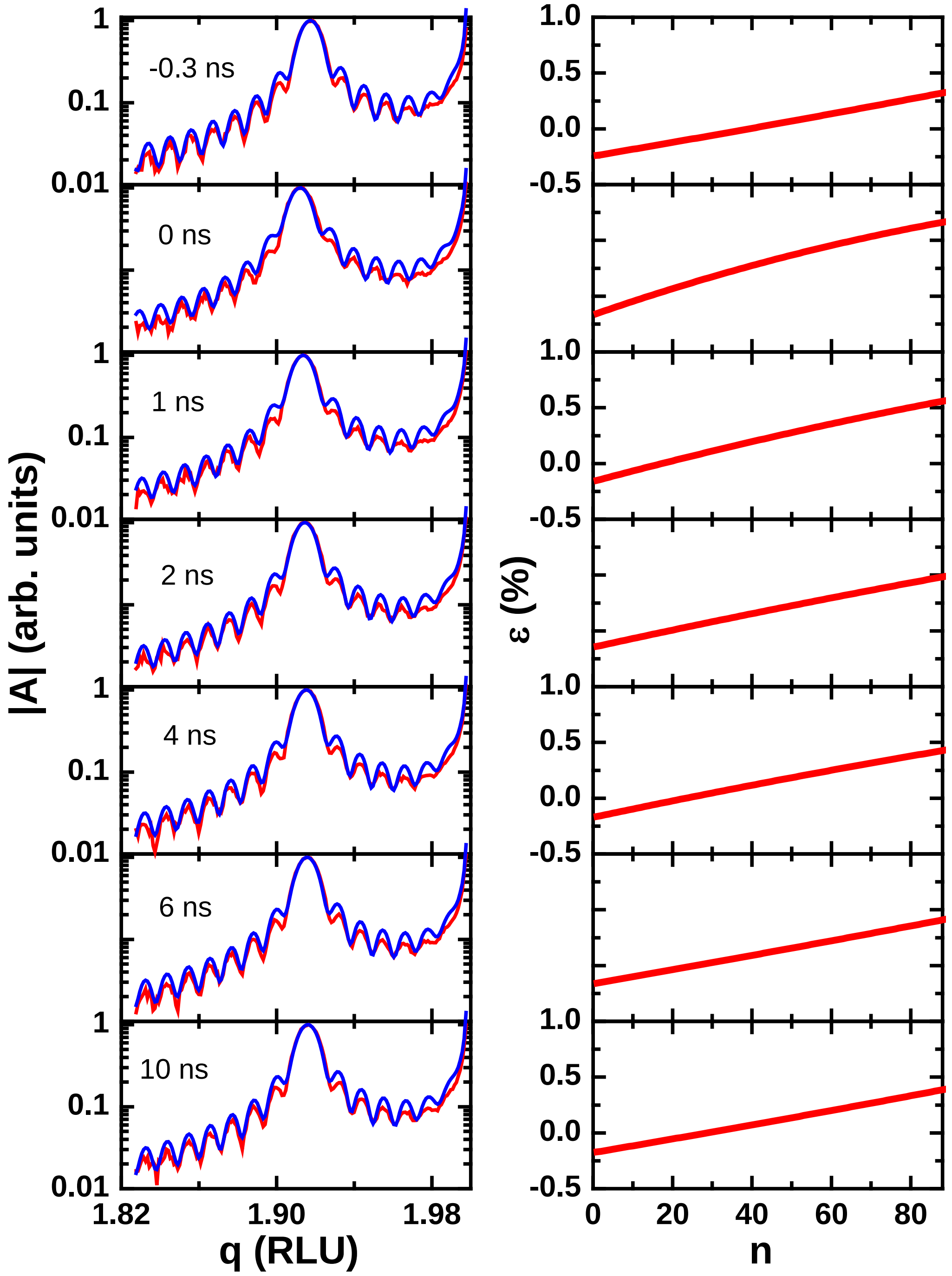

Figure S2 (a) A complete data set of the strain as a function of delay for a 35 nm film (88 monolayers) at 2.5 mJ/cm^2. Left column: measured (red lines) and fitted (blue lines) diffraction amplitude |A| at different delays between the laser and the X-ray. Left column: corresponding fitted strain at different delays as a function of the film layer index n.

`

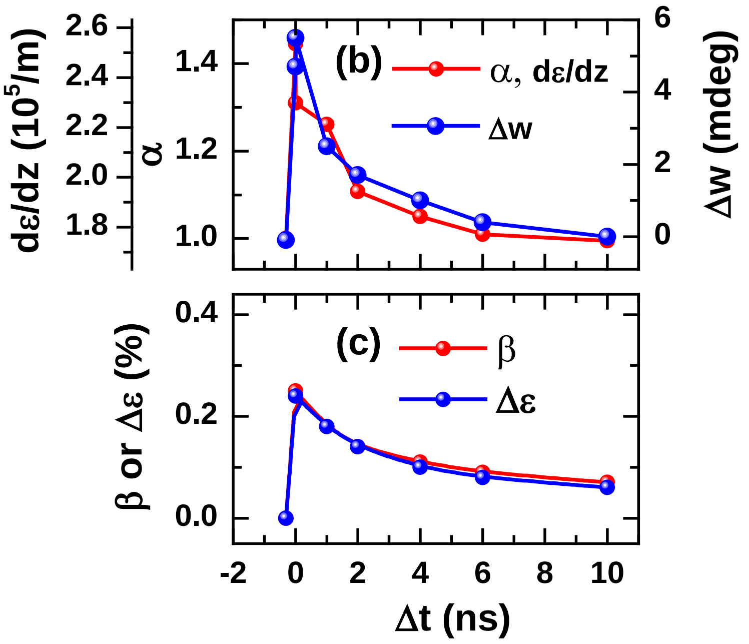

Figure S2 (b) Fitting parameters $\alpha(t)$ and the corresponding strain gradient $d\varepsilon/dz$ in Eqs.(1) (red) in comparison with measured width change $\Delta w(t)$ (blue). (c) Parameter $\beta(t)$ in Eq. (1) (red) and average strain change $\Delta\varepsilon(t)$ (blue).

`

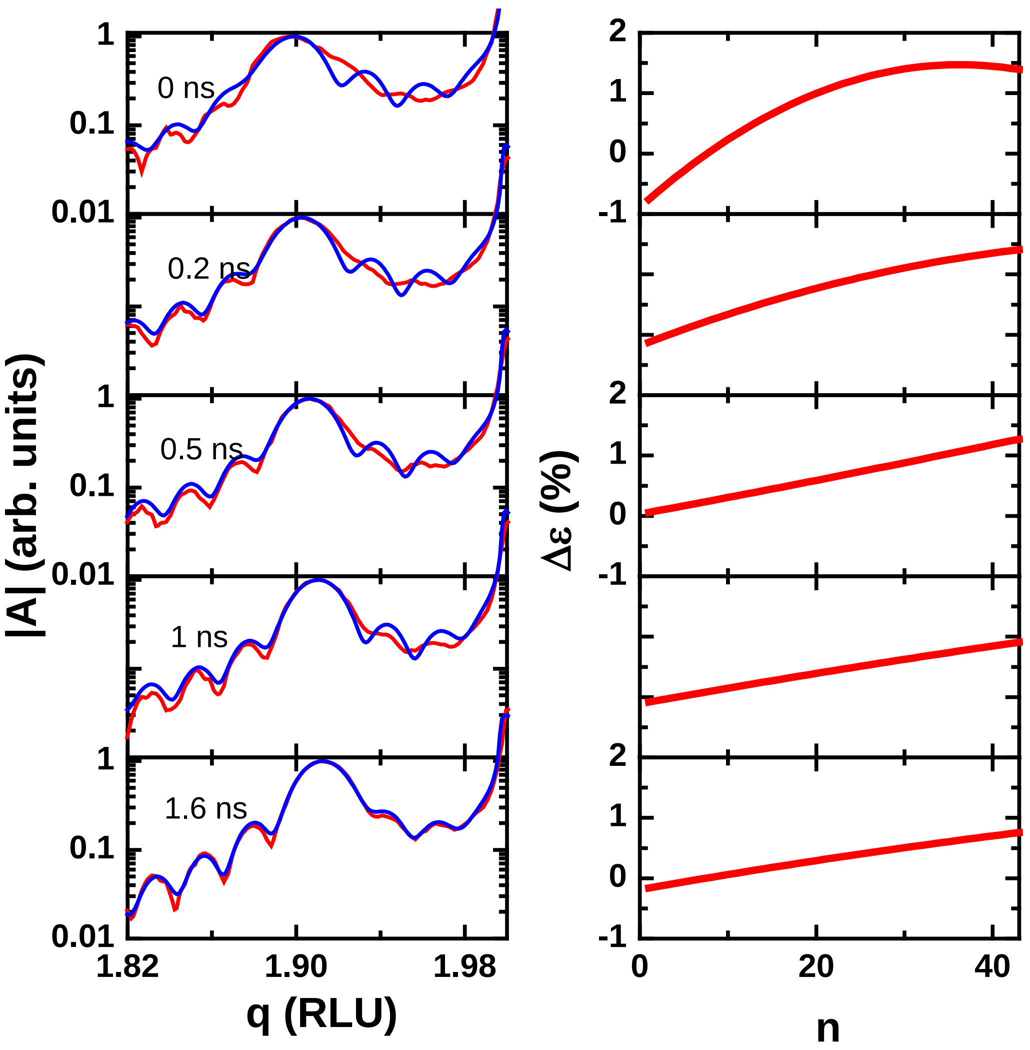

Figure S3 (a) A complete data set of the mapping of the strain as a function of delay for a nominal 20 nm film (50 monolayers) at 3.3 mJ/cm^2. Left column: measured (red lines) and fitted (blue lines) diffraction amplitude |A| at different delays between the laser and the X-ray. Left column: corresponding fitted strain at different delays as a function of the film layer index n. The fitting is very good for all delays except for zero delay.

`

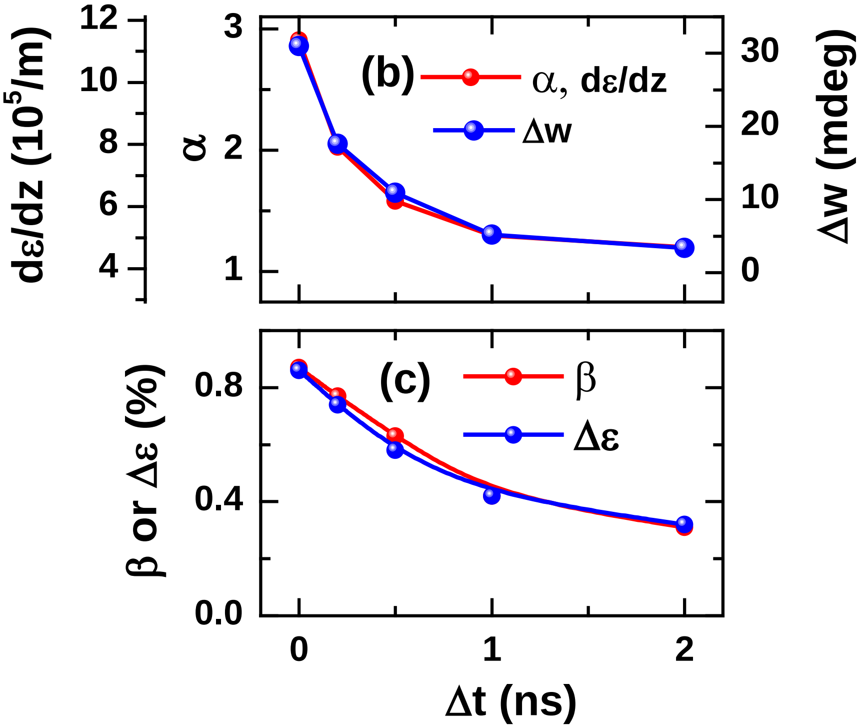

Figure S3 (b) Fitting parameters $\alpha(t)$ and the corresponding strain gradient $d\varepsilon/dz$ in Eqs.(1) (red) in comparison with measured width change $\Delta w(t)$ (blue). (c) Parameter $\beta(t)$ in Eq. (1) (red) and average strain change $\Delta\varepsilon(t)$ (blue).

`

Supplementary Information 2

DFT simulation

DFT calculations were performed in QUANTUM ESPRESSO[2] using the local density spin approximation[3,4] and an on-site Coulomb parameter[5] of $U = 4$ eV applied to the Fe states[6]. The Fe atoms were initialized to an antiferromagnetic spin configuration. Vanderbilt ultrasoft pseudopotentials[7] with scalar relativistic corrections simulated the core and valence electrons. The pseudopotentials were generated with the Perdew-Zunger parameterization[8] of the local density approximation in DFT using the following parameters: Bi: $6s^2 5d^{10} 6p^3$, r_0=1.2 bohr, r_{loc}=2.2 bohr, r_c = (2.5,2.5,2.2) bohr for *s*, *p*, and *d*, respectively. Fe: $3s^2 3p^6 4s^2 3d^6 4p^0$, r_0=1.5 bohr, r_{loc}=2.0 bohr, r_c = (2.0,2.0,2.0) bohr for *s*, *p*, and *d*, respectively. O: $2s^2 2p^4$, r_0=0.7 bohr, r_{loc}=1.0 bohr, r_c = (1.2,1.2) bohr for *s* and *p*, respectively. A plane wave basis supporting the wave function (density) cutoff at 50 Ry (400 Ry) converged the rhombohedral $R3c$ and cubic perovskite $Pm\bar{3}m$ structural energy difference to within 1 meV per formula unit. A Γ-centered 5×5×5 Monkhorst-Pack k-point mesh for the $R3c$ structure sampled the Brillouin zone to converge total energy to within 10 meV per function unit. The sum of the forces on the ions was relaxed in the $R3c$ symmetry to less than 20 meV/angstrom, and the pressure on the simulation cell in the same symmetry to less than 0.01 kbar. A Fermi-Dirac distribution applied to the occupation of the DFT single-particle states[9] simulated the effect of excitation by varying the width of the distribution from 0.00 eV up to 2.04 eV to increase electron temperature. The gap between the highest occupied state and the lowest unoccupied DFT single-particle state disappears between Fermi-Dirac distribution widths 0.95 and 1.08 eV. The relaxed spin state transitions from antiferromagnetic to nonmagnetic between Fermi-Dirac distribution widths of

`

1.36 and 1.50 eV. Bi and Fe ions retain a presumably ferroelectric displacement from centrosymmetric positions with $R\bar{3}c$ symmetry at all Fermi-Dirac distribution widths simulated.

The rhombohedral lattice parameters were then converted to pseudo-cubic. The out-of-plane strain was calculated according to Hooke's law, taking into account the coupling of epitaxial in-plane stress due to the cold substrate:

$$\varepsilon = \frac{2\nu}{1-\nu}\varepsilon_{in}, \quad \text{(s3)}$$

where $\nu = 0.34$ is the Poisson ratio[10], and ε and ε_{in} are the out-of-plane and in-plane strain, respectively.

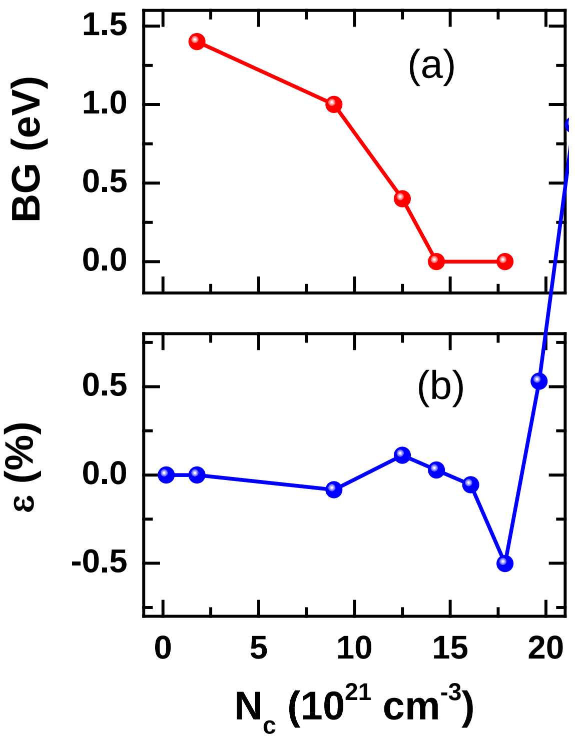

Figure S4 Kohn-Sham band gap (BG) (a) and out-of-plane strain (b) as a function of the carrier density (N_c) from DFT simulations. The strain calculation takes into account the Poisson effect due to the in-plane stress. The N_c corresponding to the highest pump fluence of 5 mJ cm^{-2} in the experiment is about 1.5×10^{21} cm^{-3}.

`

Supplementary Information 3

Dependence of the piezoelectric coefficient on the strain

From Eqs. (2 -4), we have

$$\alpha(t) - 1 = \gamma d_0 E_{scr}(t). \quad \text{(s4a)}$$

$$\gamma = \frac{\alpha(t)-1}{E_{scr}d_0} = \frac{\alpha(t)-1}{\beta(t)-\varepsilon_{scr}(t)}. \quad \text{(s4b)}$$

The resulting γ using our experimental data is shown in Table S1.

Table S1. Parameter γ obtained from the experimental data. The errors are due to the uncertainty in determining the thermal contribution and the uncertainty in the fitting.

	20 nm, 3.3 mJ cm^{-1}	35 nm, 3.3 mJ cm^{-1}	35 nm, 2.5 mJ cm^{-1}
Delay (ns)	0.2	0	0
α	1.97	1.57	1.38
β	0.76%	0.37%	0.26%
ε_h	0.16%	β/3	β/3
γ	160±50	234±80	216±70

`

Supplementary Information 4

Disproving the charge separation by polarization field model

Bulk charge separation due to internal polarization or external field leads to strong distortion of the field in a pump probe experiment though it may have no effect in a CW experiment. We simulated the situation with a one dimensional particle-in-cell dynamic model for the 35 nm film where the motion of the carriers (Fig. S4(a)) and the field (Fig. S4(b)) are solved self-consistently. Equal number of holes and electrons are generated filling the space with a profiled probability $p(z)=\exp(-z/Z)$, where Z = 32 nm is the absorption length at 400 nm. To illustrate the physics, we apply a constant field E_0 = 1 MV/cm that generates a peak strain of 0.5% at a nominal piezoelectric constant d_{33} = 50 pm/V. We use nominal mobility for the electrons and holes of 7×10^{-5} and 5×10^{-5} $m^2V^{-1}s^{-1}$, respectively, for illustrative purpose. The result is not sensitive to the choice of these numbers other than the time scale. A dielectric constant of 50 is used[11]. The carriers are absorbed when they reach the boundaries. We also consider only the cases where carriers are immediately separated at birth.

For carrier density higher than 3×10^{18} cm^{-3} (corresponding roughly to an absorption fluence of 0.01 mJ/cm^2), 100% modulation of the field can be achieved, indicating the saturation of the applied field. For lower carrier densities, the maximum modulation is proportional to the carrier density. Fig. S3 shows a case with carrier density of 1.5×10^{18} cm^{-3} (absorption fluence of 0.005 mJ/cm^2) As can be seen in Figure S3 (b), the charge separation induces a ±50% modulation of the field inside the film. When mapped into the piezoelectric response of the unit cells, the modulation will lead to a strain profile completely different from that arises from a uniform field expected from the exciton scenario where carriers only separate at the surface and

`

the interface. The low carrier density needed for such modulation also demonstrates the sensitivity of the strain profile to such a bulk space charge effect. Simulation using strain profile following the field profiles with comparable strain range as measured from the experiment generates significantly asymmetric fringes around the central diffraction peak, which is not observed in the experimental data (Fig. S5), disproving the commonly accepted carrier separation by polarization field model.

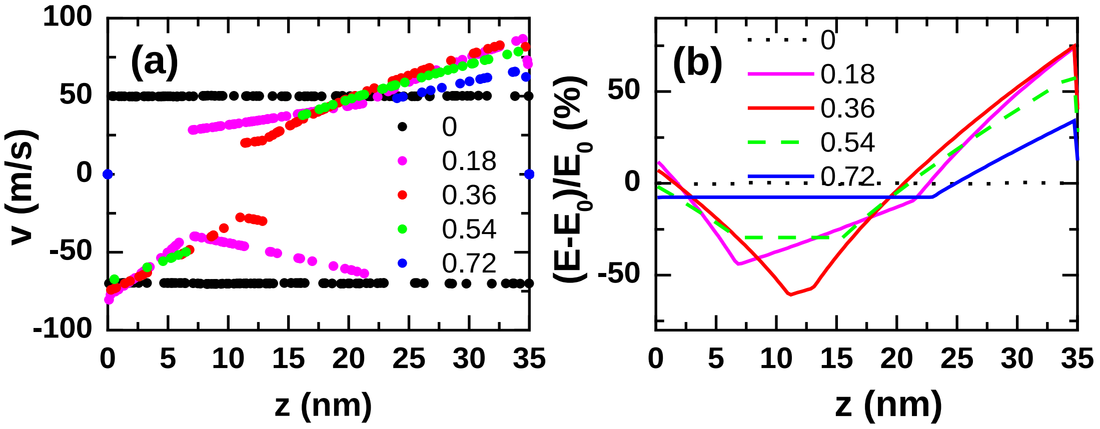

Figure S5 Effect of the field inside a 35 nm film when the carriers are generated by an impulsive excitation and immediately separated by an external field. (a) Position-velocity phase diagram of the holes (positive speed) and electrons (negative speed) at different times (indicated by the legend in ns) and (b) the corresponding field modulation as the carriers are separated by the applied field of E_0=1 MV/cm. The initial carrier density is 1.5×10^{18} cm^{-3} corresponds roughly to an absorption fluence of about 0.005 mJ/cm^2, less than 1% of the that used in our experiment. The asymmetry in the field and phase diagram are due to the difference in the electron and hole mobility.

`

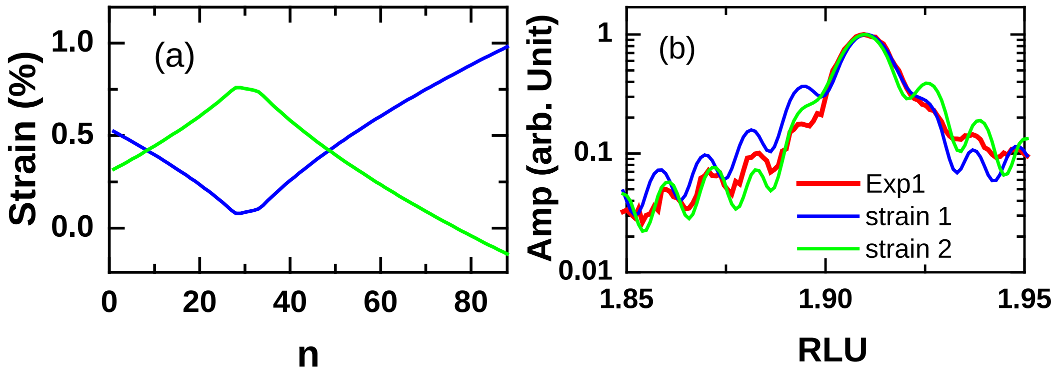

Figure S6 Simulation of the diffraction pattern using strain profile derived from the field distribution in Fig. S4. (a) Two possible strain profiles and (b) the simulated diffraction patterns near the 0 0 2 diffraction peak in comparison with the experiment measurement for the 35 nm high fluence case. To obtain the strain profile, we map the field profile to the strain range observed in the experiment with a shift to match the diffraction peak position. A strong fringe intensity asymmetry is predicted but not observed in the experiment. This confirms the quality of our fit in Figs. 1, S1-S3 and the interpretation of the data, disproving the commonly accepted carrier separation by polarization field model.

`

Supplementary references

1. Boulle, A., Masson, O., Guinebretière, R. & Dauger, A. A new method for the determination of strain profiles in epitaxic thin films using X-ray diffraction. *J. Appl. Crystallogr.* **36,** 1424–1431 (2003).
2. Giannozzi, P. *et al.* QUANTUM ESPRESSO: a modular and open-source software project for quantum simulations of materials. *J. Phys. Condens. Matter* **21,** 395502 (2009).
3. Hohenberg, P. Inhomogeneous Electron Gas. *Phys. Rev.* **136,** B864–B871 (1964).
4. Kohn, W. & Sham, L. J. Self-Consistent Equations Including Exchange and Correlation Effects. *Phys. Rev.* **140,** A1133–A1138 (1965).
5. Anisimov, V. I., Aryasetiawan, F. & Lichtenstein, A. I. First-principles calculations of the electronic structure and spectra of strongly correlated systems: the **LDA** + *U* method. *J. Phys. Condens. Matter* **9,** 767–808 (1997).
6. Neaton, J., Ederer, C., Waghmare, U., Spaldin, N. & Rabe, K. First-principles study of spontaneous polarization in multiferroic $BiFeO_3$. *Phys. Rev. B* **71,** 014113 (2005).
7. Vanderbilt, D. Soft self-consistent pseudopotentials in a generalized eigenvalue formalism. *Phys. Rev. B* **41,** 7892–7895 (1990).
8. Perdew, J. P. Self-interaction correction to density-functional approximations for many-electron systems. *Phys. Rev. B* **23,** 5048–5079 (1981).
9. Baroni, S., de Gironcoli, S. & Dal Corso, A. Phonons and related crystal properties from density-functional perturbation theory. *Rev. Mod. Phys.* **73,** 515–562 (2001).
10. Biegalski, M. D., Dörr, K., Kim, D. H. & Christen, H. M. Applying uniform reversible strain to epitaxial oxide films. *Appl. Phys. Lett.* **96,** 151905 (2010).

`

11. Catalan, G. & Scott, J. F. Physics and Applications of Bismuth Ferrite. *Adv. Mater.* **21,** 2463–2485 (2009).
